# A Novel Rheological Method to Assess Drug-Polymer Interactions Regarding Miscibility and Crystallization of Drug in Amorphous Solid Dispersions for Oral Drug Delivery

**DOI:** 10.3390/pharmaceutics11120625

**Published:** 2019-11-22

**Authors:** Georgia Tsakiridou, Christos Reppas, Martin Kuentz, Lida Kalantzi

**Affiliations:** 1 Department of Scientific Affairs, Pharmathen S/A, 15125 Marousi, Greece; gtsakiridou@pharmathen.com; 2 Department of Pharmaceutical Sciences, National and Kapodistrian University of Athens, 15784 Zografou, Greece; reppas@pharm.uoa.gr; 3 Institute of Pharmaceutical Technology, University of Applied Sciences and Arts Northwestern Switzerland, 4132 Muttenz, Switzerland; martin.kuentz@fhnw.ch

**Keywords:** amorphous solid dispersions, polymer-API miscibility, rheology, Hansen solubility parameters

## Abstract

Solid dispersions provide a key technology to formulate poorly water-soluble drugs, and a main task of early development is appropriate selection of polymer. This study investigates the use of a novel rheology-based approach to evaluate miscibility and interactions of drugs with polymers regarding amorphous solid drug dispersions for oral administration. Tacrolimus was used as model drug and hydroxypropyl cellulose, ethylcellulose, Soluplus^®^, polyethyleneglycol 6000, Poloxamer-188 (Koliphor-188), and Eudragit^®^ S100 were used as excipients. Solvent-based evaporation methods were used to prepare binary solid dispersions of drug and polymer. Data of the dilute solution viscosimetry were compared with in silico calculations of the Hansen solubility parameter (HSP), as well as phase separation/crystallization data obtained from X-ray diffraction and differential scanning calorimetry. HSP calculations in some cases led to false positive predictions of tacrolimus miscibility with the tested polymers. The novel rheology-based method provided valuable insights into drug-polymer interactions and likely miscibility with polymer. It is a rather fast, inexpensive, and robust analytical approach, which could be used complementary to in silico-based evaluation of polymers in early formulation development, especially in cases of rather large active pharmaceutical ingredients.

## 1. Introduction

In recent years, the pharmaceutical industry is pursuing less druggable targets, with characteristics such as increased size and flexibility [1,2]. Especially, the molecular weight of active pharmaceutical ingredients (APIs) shows a steady increase over the years. Currently, most APIs have a molecular weight of 300–400 g/mol, but the projection is that the pharmaceutical industry will be dealing with relatively bigger APIs (>500 g/mol) in the future [1]. This general shift in API characteristics has spurred an interest in enhanced oral formulation technologies of such molecules [3,4,5], with amorphous solid dispersions (ASD) as a key approach to improve apparent solubility and oral bioavailability of APIs with the above-mentioned characteristics [6,7,8].

A variety of options exist for the preparation of ASDs, including the most widespread manufacturing by spray drying and hot melt extrusion (HME) [9]. However, independent of the preparation method, it is important to stabilize the amorphous state during the shelf life of the drug product, as well as to sustain supersaturation during drug release. Even though other types of solid dispersions exist [10], polymer-based systems are the most frequently employed; polymers can stabilize the amorphous API by ensuring adequate API dispersion in the matrix, by increasing the glass transition temperature (*T*_g_) of the API, and/or by kinetic entrapment of the API in the polymer matrix [6,11]. Polymers can, moreover, inhibit re-crystallization during dissolution, especially for poor glass-forming APIs and those with low particle/cluster size, which have been linked to a tendency for re-crystallization due to the high mobility of the molecules on the surface of ASDs [12,13]. Generally, in polymer-based ASDs, a critical initial step is the identification of appropriate API-polymer combinations which will likely lead to long term stabilization of the dispersed amorphous drug phase. Relevant evaluations are based on the miscibility characteristics of the drug with the polymer [14].

Miscibility screening of active pharmaceutical ingredients with various polymers has been troubling formulation scientists for a long time in search of a more efficient way to cope with the experimental work load [15]. Usually, lab-scale solid dispersions are prepared via techniques such as solvent shift [16,17], solvent evaporation, film casting [18,19], and single droplet drying [20], which can be seen as the small-scale equivalent to the industrial process of spray drying. Phase separation and crystallization are subsequently assessed by solid state characterization techniques, including thermal methods (differential scanning calorimetry (DSC), melting point depression, glass transition temperature calculation), spectroscopic techniques (Fourier transform infrared spectroscopy (FTIR), solid-state nuclear magnetic resonance (ssNMR), X-ray powder diffraction (XRPD)), and microscopic imaging techniques (scanning electron (SEM) and transmission electron microscopy (TEM), polarized light microscopy, atomic force microscopy (AFM), and Raman imaging). Miscible combinations tend to remain homogenous, with the amorphous API being adequately dispersed in the polymer matrix. On the other hand, immiscible combinations will separate into drug-rich and drug-poor phases, with the drug-rich regions being more susceptible to recrystallization as there is not enough polymer to stabilize amorphous API over time [21,22].

Miniaturized assays have been developed to cope with the demands of workload and drug consumption associated with the study of drug-polymer interactions, together with the miscibility characteristics [23]. However, these assays require sophisticated equipment and, even though automation is a prospect, it is still not well established in the pharmaceutical industry as extensive optimization is needed for each API-polymer combination. Other approaches have focused on estimating the drug solubility in polymer, which can also guide selection of polymer in ASDs similar to any miscibility assessment, and an interesting method has been reported by Knopp et al. [24].

In silico approaches to screen excipients regarding stable ASDs have been also proposed, in order to minimize time and material consumption [25]. The preferred choice would be modern thermodynamic approaches, but, currently, it is challenging to obtain the required parameters (chemical molecular descriptors, such as specific volume, activity coefficient, etc.) for the modeling of pharmaceutical formulations [26,27]. Today, simpler thermodynamic approaches based on, for example, the classical Flory-Huggins chi interaction parameter (*χ*), are used to assess the miscibility of drug-polymer blends [28]. Low values of *χ* (<0.5) indicate that adhesive forces are sufficiently high compared to cohesion to enable drug-excipient miscibility [29]. However, the *χ* interaction parameter shows limitations in predicting miscibility for molecules that form specific interactions, such as hydrogen bonding [30], as it primarily accounts for non-specific dispersion forces [31]. Another rather simple thermodynamic approach is the use of the total solubility parameter (*δ*) [32,33], where similarity between values of drug and polymer would suggest miscibility [34,35]. The solubility parameter can be further used to calculate the *χ* interaction parameter [31]. However, any single interaction value may not sufficiently reflect the different kinds of molecular interactions. More promising is here the use of partial solubility parameters (e.g., the Hansen solubility parameters) to differentiate between dispersive, polar, and hydrogen bonding interactions [36]. There are still several theoretical limitations reported, which can be viewed as a downside of the given simplicity [37,38]. Partial solubility parameters are still useful, especially when they are predicted in silico, as the otherwise experimental determination is quite time consuming and often not practical in early formulation development [39].

Attempting to find a cost effective, less labor-intensive method of excipient miscibility assessment for ASDs, we evaluated a novel rheological characterization of diluted drug-polymer solutions. Dilute solution viscosimetry has been used extensively in the investigation of polymer-polymer miscibility in polymer blends. Dilute polymer solutions enable study of polymer chain interactions as a solution approach, and theoretically, ideal solutions are approximated in which, for example, intramolecular interactions are kept to a minimum. This methodology is based on the notion that intrinsic viscosity measurements of polymer blends represent the dimension of polymer coils that expand or retract, depending on whether interactions are attractive or repulsive [40]. Different ways exist to study dilute solution viscosimetry, and such an approach has been previously employed in the estimation of solubility parameters [41,42,43,44,45]. A very recent study presented a miniaturized rheological method to investigate drug-polymer solutions that reflected molecular interactions relevant for the in vitro performance of ASDs [46]. Such miniaturization should advance the use of dilute solution viscosimetry in early formulation development in general. One such approach can be described as the data treatment by Chee [47], which has been proved promising for hydrogels [48,49]. In this methodology, intrinsic viscosity measurements are translated to viscometric interaction parameters that can reveal overall interactions between two molecules in solution, especially when dealing with large molecules such as polymers. In addition, the miscibility outcome from this method can be extrapolated to the solid state [50,51,52], making the use of a variety of preparation methods possible.

**Figure 1:**
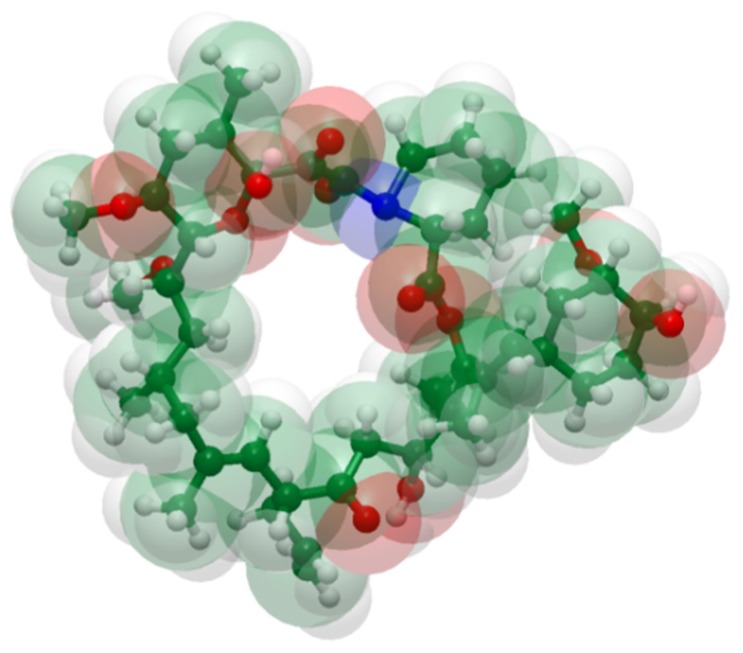
Structure of tacrolimus from a space-filling model using standard color codes of the elements (white is for Hydrogen, green for Carbon, red for Oxygen and blue for Nitrogen atoms).

We therefore evaluate the rheological method of Chee [47] for its usefulness in the screening of ASDs comprising polymer and the model drug, tacrolimus (Figure 1), which is a rather large molecule. Relevant data are discussed in view of in silico estimations of the Hansen solubility parameter (HSP), as well as phase separation data obtained from XRPD and DSC, wherein ASDs were prepared by solvent evaporation methods.

## 2. Materials and Methods

### 2.1. Materials

Crystalline tacrolimus was purchased from Apotex (Toronto, ON, Canada). Hydroxypropyl cellulose (HPC-L, Klucel™) was purchased from Ashland (Covington, KY, USA) and Ethylcellulose (EC, Ethocel STD 10 Premium) was purchased from Colorcon Inc.^®^ (Harleysville, PA, USA). The polyethylene glycol, polyvinyl acetate and polyvinylcaprolactame-based graft copolymer (Soluplus^®^), and the poly(ethylene oxide)-poly(propylene oxide)-poly(ethyelene oxide) triblock copolymer (Poloxamer-188, Kolliphor P-188) were purchased from BASF (Ludwigshafen, Germany). Polyethylene glycol 6000 (PEG 6000) was purchased from Clariant AG (Muttenz, Switzerland) and the methacrylic acid-methyl methacrylate copolymer (Eudragit^®^ S100) was purchased from Evonik Industries AG (Essen, Germany). Ethanol was purchased from Honeywell Research Chemicals (Morris Plains, NJ, USA). All excipients and chemicals were acquired from commercial sources and were used as obtained. In Table 1, the physicochemical characteristics of the compounds used in this study are described.

### 2.2. Methods

#### 2.2.1. Preparation of Tacrolimus-Polymer Solid Dispersions

##### Rotary Evaporation

Approximately 2 g of each dry tacrolimus-polymer blend was dissolved in 250 mL of ethanol. Six tacrolimus-polymer combinations were used, including the combinations of tacrolimus with HPC-L, EC, Soluplus^®^, PEG-6000, Poloxamer-188, and Eudragit^®^ S100, with two levels of tacrolimus loading (10% and 90%). The dissolved blends were transferred to flasks for rotary evaporation in a STEROGLASS^®^ evaporator (STRIKE 100) (Perugia, Italy). The temperature of the water bath was kept constant at 70 °C and the rotation was set at 50 rpm. Vacuum was applied to facilitate the evaporation of ethanol. After approximately 1.5 h, the solvent was removed, and the solid mass was scraped off the glass flask and stored in sealed containers at 25 °C and 40 °C. Yield was variable and was estimated around 50–70% (*w*/*w*) due to material consistency after drying.

##### Film Casting

Approximately 2 g of each of the six tacrolimus-polymer blends with two drug loadings (10–90%, *w*/*w*) were dissolved in 100 mL of ethanol and were placed on petri dishes (SARSTEDT, Nümbrecht, Germany). The petri dishes were then placed in an STF F120 drying oven (FALC, Treviglio, Italy) with a constant temperature of 70 °C for drying over 24 h. The final material was a soft or crispy film, depending on drug loading, and it was stored in sealed containers at 25 °C and 40 °C.

The tacrolimus solid dispersions prepared with each of these methods were stored in closed containers and stored in stability chambers for stability testing at temperatures of 25 ± 2 °C (ENVIMED WALK-IN-16, Bangkok, Thailand) and 40 ± 2 °C (Memmert THPP 749, Büchenbach, Germany).

#### 2.2.2. Characterization of the Physical Drug State

The physical state of tacrolimus in the solid dispersions was subsequently tested using XRPD and DSC at time zero, as well as after 1 and 3 months by means of DSC. Containers remained closed in the stability study, because an effect of moisture-induced phase separation and crystallization was not the focus of this study.

##### Differential Scanning Calorimetry

Samples were weighted to 5 ± 1 mg and placed to 50 μL aluminum pans (Perkin-Elmer, Hopkinton, MA, USA). The pans were placed on the heat plate of a Perkin-Elmer DSC 6 (Perkin-Elmer, MA, USA) and they were heated from 0 to 170 °C at a rate of 10 °C/min, under a constant flow rate of nitrogen at 40 mL/min. An empty pan was used as reference. The thermodynamic events of the samples were monitored using the Pyris software (Perkin-Elmer, MA, USA).

##### X-ray Powder Diffraction

A Rigaku MiniFlex 600 diffractometer was used (Rigaku, New Trails Dr, The Woodlands, TX, USA) for the XRPD measurements. The diffractometer was equipped with a Cu anode using a Kα1 radiation source and a small scintillation counter detector with a Kβ filter. A range of 2θ from 5 to 40° was scanned using a detector step width of 0.02° and a measurement time of 1.5 s per step; total measurement time was 57 min. The absence of discernable peaks and the presence of a “halo” indicate amorphous material.

#### 2.2.3. In Silico Approach

##### Calculation of Hansen Solubility Parameters (HSPs)

HSPs were originally experimentally determined via solubility experiments [53]. However, the development of group contribution theories provided the opportunity for predicting HSPs based on the chemical structure of the materials. Group contribution methods fragment a molecular structure, and functional groups are assigned to values contributing to, for example, cohesive energy. A variety of group contribution approaches have been proposed over the years [54,55,56,57].

HSPs in this study were calculated via the Y-MB group contribution method using the Hansen Solubility Parameters in Practice (HSPiP) software (Hansen Solubility Parameters in Practice software Version 5.0.06, 2008–2017, www.hansen-solubility.com, London, UK). Compound structures were generated using the Chem Draw Ultra Version 12.0.2.1076 (Perkin-Elmer, MA, USA) molecular builder and they were used as input to the HSPiP software.

The solubility parameter rule of thumb proposed by Greenhalgh et al. was used to assess tacrolimus-polymer miscibility in this study. Based on that proposal, two compounds are deemed immiscible if the difference in the solubility parameters (Δ*δ_t_*) is greater than 10 MPa^0.5^. A difference that is less than 7 but greater than 2 MPa^0.5^ suggests miscibility, and a difference of less than 2 MPa^0.5^ may indicate a solid solution [34].

##### Construction of Hansen Solubility Spheres

Miscible and immiscible combinations can be further represented in the context of Hansen solubility spheres. HSPs can be used in plots of the different partial solubility parameters in terms of polar (*δ_p_*), dispersive (*δ_d_*), and hydrogen bonding (*δ_h_*) contribution [33,42,53,58], where the distance, *R_a_,* between compounds 1 and 2 is given by Equation (1):

(1) $$R_{a}{}^{2}=4\;{\left(\delta_{d1}-\delta_{d2}\right)}^{2}+{\left(\delta_{p1}-\delta_{p2}\right)}^{2}+\;{\left(\delta_{h1}-\delta_{h2}\right)}^{2}$$

For an assumed miscible combination, the distance *R_a_* between any two materials should not exceed a maximum value, i.e., the radius of interaction *R*_0_. This radius is usually calculated by identifying “good” and “bad” solvents with known partial solubility parameters for the material of interest [59]. The ratio of *R_a_/R*_0_ is called relative energy difference (RED) and can be seen as an indicator of whether a compound is likely to exist within the solubility sphere of the investigated material. An RED value greater than 1 indicates that the distance between two materials exceeds the maximum *R*_0_ value, and thus, they are likely not miscible. On the other hand, an RED value of less than 1 can be seen as a positive indication for miscibility.

The *R*_0_ value for tacrolimus was calculated based on the partial solubility parameters data in the library of the HSPiP software, which has been experimentally validated [60].

#### 2.2.4. Rheology Studies

In order to calculate the interaction parameters, the reduced viscosities of the blend solutions, as well as the solutions of each compound alone, were measured. Ethanol was used as solvent to prepare solutions of tacrolimus, each individual polymer, as well as blends of polymer with different drug loadings of 10, 25, and 50% (wt %). Higher drug loadings were not used, as the rheological behavior of the solutions would resemble the pure API solution, making it difficult to characterize tacrolimus-polymer interactions. Reduced viscosities (*n_red_*) have traditionally been measured with a glass Ubbelohde viscometer, where the time that a solution takes to pass between two graded lines on the viscometer can be translated to viscosity. The time for a solution (*t*) is then divided by the time for the used solvent (*t*_0_), giving the relative viscosity (*η_rel_*) of the solution (Equation (2)). The relative viscosity is used to calculate the reduced viscosity (dL/g) by Equation (3), where *C* is the total concentration of the solution (tacrolimus and polymer, g/dL).

(2) $$\eta_{rel}=\frac{t}{t_{0}}$$

(3) $$\eta_{red}=\frac{\eta_{rel}-1}{C}$$

Reduced viscosity cannot be considered a viscosity or a pure number, but rather, a traditional characteristic named in polymer science. The reduced viscosities of each solution in at least five dilutions (0.1, 0.2, 0.3, 0.4, and 0.5 wt %) are plotted against the concentrations and a linear graph is acquired. According to Chee’s model from this graph, the intercept is the intrinsic viscosity [*η*] (dL/g) and the slope corresponds to the parameter *b*. Then, an interaction parameter can be calculated as a function of *b* according to Equation (4):

(4) $$\Delta B=\frac{b-\;\bar{b}\;}{2w_{1}w_{2}}$$

where *b* is calculated by Equation (5) and $\bar{b}$, which is the *b* average, by Equation (6).

(5) $$b=w_{1}{}^{2}b_{11}+\;w_{2}{}^{2}b_{22}+2w_{1}w_{2}b_{12}$$

(6) $$\bar{b}=w_{1}b_{11}+\;w_{2}b_{22}b_{11}$$

where *b*_11_ and *b*_22_ are the slopes from the reduced viscosity curves from the solutions of the pure compounds, and *w*_1_ and *w*_2_ are the weight fractions for the drug and the polymer, respectively. The slope *b*_12_ is given from the reduced viscosity graph for the blend solution. This parameter *b* is also related to the Huggins’ coefficient *K_h_* and [*η*] the intrinsic viscosity of the solution (Equation (7)):

(7) $$b=K_{h}\eta^{2}$$

The intrinsic viscosity is an approximation at infinite dilution and holds for a solute’s contribution to the viscosity [61].

In the case that the two intrinsic viscosity values for the two different compound solutions are apart, a *μ* interaction parameter is used as a more robust evaluation option (Equation (8)), where *η*_1_ and *η*_2_ are the intrinsic viscosities of the pure compound solutions [46].

(8) $$\mu =\frac{\Delta B}{{\left(\eta_{2}-\eta_{1}\right)}^{2}}$$

When Δ*B* and *μ* are positive or equal to zero, the blends are deemed miscible, while they are considered immiscible when values are negative.

Following this initial theoretical work, Sun et al. proposed the parameter *α* (Εquation (9)), where *K*_1_, *K*_2_, and *K*_12_ are the Huggins’ coefficients for the individual components 1 and 2 and for the blend, respectively [62]. The parameter α holds more predictive power as it based on more accurate calculations [47,48,49].

(9) $$\alpha =K_{12}-\frac{K_{1}{\eta_{1}}^{2}w_{1}{}^{2}+\;K_{2}{\eta_{2}}^{2}w_{2}{}^{2}+2\sqrt{K_{1}K_{2}\eta_{1}\eta_{2}w_{1}w_{2}}}{{\left(\eta_{1}w_{1}+\eta_{2}w_{2}\right)}^{2}}$$

## 3. Results

### 3.1. Characterization of Tacrolimus-Polymer Solid Dispersions

**Figure 2:**
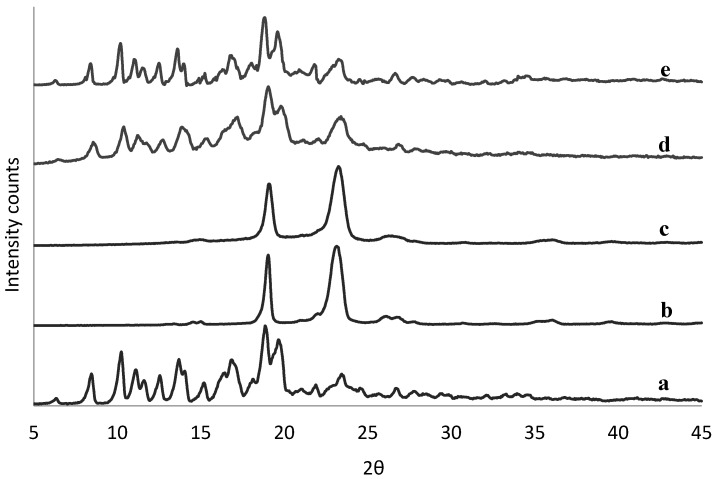
XRPD spectra of crystalline tacrolimus (**a**), Poloxamer-188 (**b**), and PEG 6000 (**c**), and of fresh tacrolimus formulations prepared via the rotary evaporator of 90% tacrolimus-Poloxamer-188 (**d**) and 90% tacrolimus-PEG 6000 (**e**).

The physical state of tacrolimus was analyzed directly following manufacture of the solid dispersions to identify potential crystallinity. There was no sign of crystallinity found in any of the solid dispersions by means of DSC (example in Figure S1). This was confirmed also by XRPD data (example in Figure S2), except for the 90% (*w/w*) tacrolimus-PEG 6000 and tacrolimus-Poloxamer-188 combinations prepared with the rotary evaporator (Figure 2) and by film casting (Figure S3).

**Figure 3:**
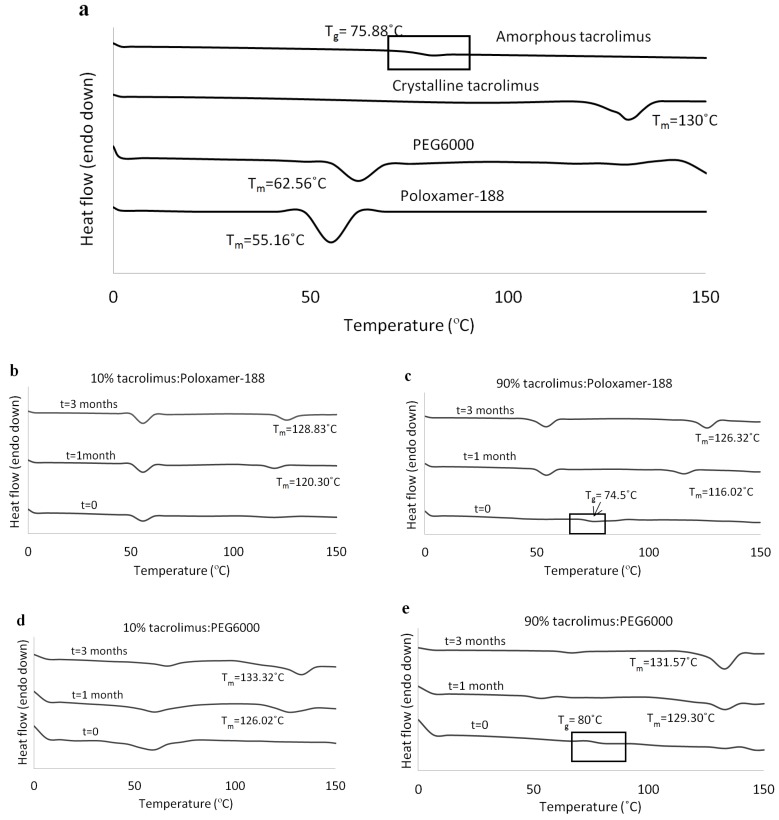
DSC thermographs for (**a**) amorphous tacrolimus, crystalline tacrolimus, PEG 6000, and Poloxamer-188, (**b**) 10% tacrolimus-Poloxamer-188, (**c**) 90% tacrolimus-Poloxamer-188, (**d**) 10% tacrolimus-PEG 6000, and (**e**) 90% tacrolimus-PEG 6000 combinations. The amorphous tacrolimus thermograph was acquired by a second heating of crystalline tacrolimus powder, and solid dispersions were prepared with the rotary evaporator.

At 1 and 3 months, tacrolimus crystallinity was investigated with DSC and the thermographs of the tacrolimus-PEG 6000 and tacrolimus-Poloxamer-188 combinations presented the characteristic melting peak of tacrolimus, irrespective of drug loading and preparation method. DSC thermographs for the solid dispersions prepared with the rotary evaporator are presented in Figure 3b–e. Similar results were acquired via the film casting method (data not shown). All other tacrolimus-polymer combinations did not show signs of drug crystallinity in the course of the stability testing (Table 2).

### 3.2. In Silico Method for the Prediction of Tacrolimus-Polymer Miscibility

HSP values were used to visualize the miscible pairs in a Hansen solubility sphere. The results, as well as the potential miscibility outcome between tacrolimus and the polymers, can be seen in Table 3 and Table 4. According to the HSP theory, tacrolimus was found to be miscible with five out of the six polymers, as shown by the HSP difference between the tacrolimus and polymer solubility parameters, which was 0.7–1 MPa^0.5^ (Table 3). Moreover, the RED value for each of the five polymers was found to be less than 1 (Table 4). However, tacrolimus was deemed immiscible with PEG 6000, because the difference of their solubility parameters was 15.6 MPa^0.5^ (Table 3) and the RED value was 1.12 (Table 4).

**Figure 4:**
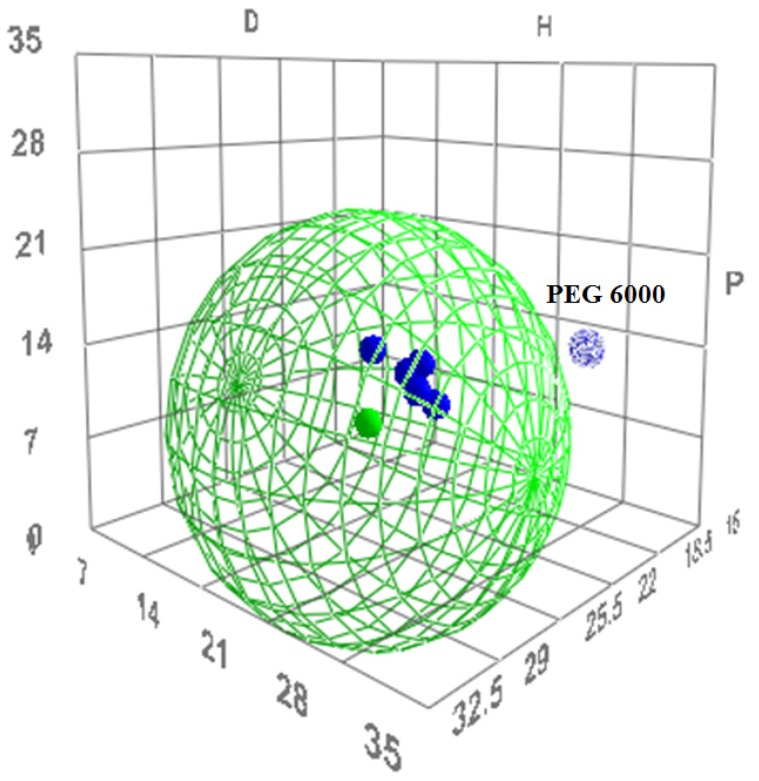
The three-dimensional (3D) evaluation of solubility parameters as depicted by the *δ_d_* (D) vs. *δ_p_* (P) vs. *δ_h_* (H) plot, as derived by the HSPiP software. Tacrolimus is depicted as a solid green dot, while the interaction radius *R*_0_ is marked by the green wire border. With blue are depicted the polymers tested. Dark blue dots represent the polymers that are expected to be miscible with tacrolimus, while the light blue dot represents the immiscible combination. The distance of each polymer from tacrolimus is calculated as the distance *R_a_* (Equation (7)).

The *R_0_* value for tacrolimus yielded 22 MPa^0.5^ and its boundaries in the Hansen solubility sphere can be seen as the green wire structure around tacrolimus in Figure 4. PEG 6000 as immiscible with tacrolimus is shown indeed outside of the boundaries given by the Hansen sphere.

### 3.3. Miscibility Investigation with the Rheology-Based Technique

**Figure 5:**
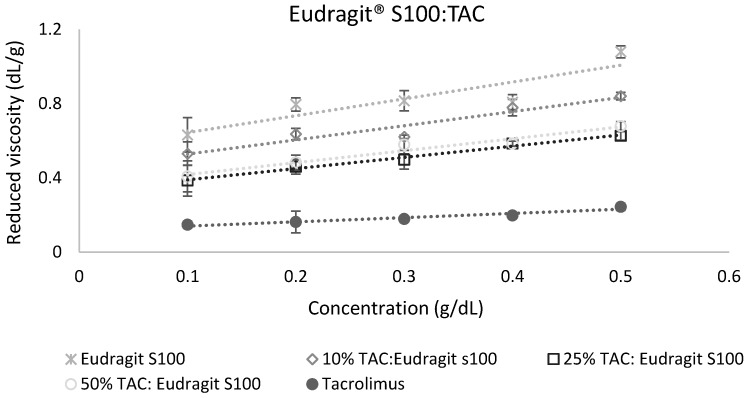
Reduced viscosity vs. concentration graph for the tacrolimus (TAC)–Eudragit^®^ S100 blend. This is an example of a miscible system (at 10% drug loading: *α* = 2.40, at 25% drug loading: *α* = 3.65, at 50% drug loading: *α* = 1.71) according to the dilute solution rheology theory.

**Figure 6:**
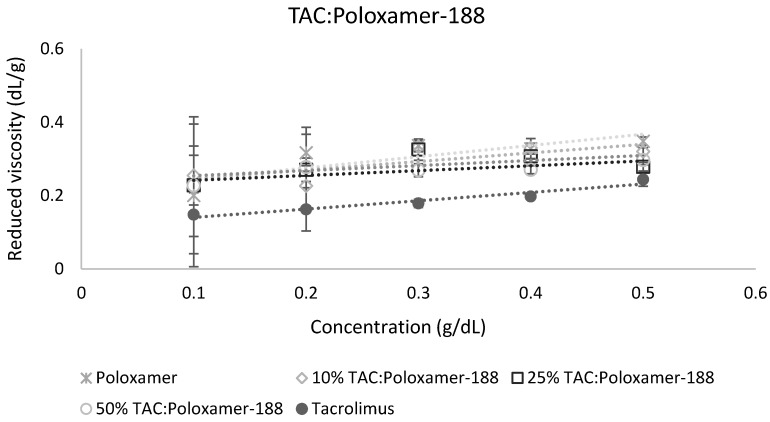
Reduced viscosity vs. concentration graph for the tacrolimus (TAC)-Poloxamer-188 blend. This is an example of an immiscible system (at 10% drug loading: *α* = −2.38, at 25% drug loading: *α* = −3.38, at 50% drug loading: *α* = −5.32) according to the dilute solution rheology theory.

The calculation of miscibility parameters was used for the estimation of tacrolimus-polymer miscibility, based on slope and intercept values of reduced viscosity vs. concentration graphs for each tacrolimus-polymer blend. Examples of these graphs are shown in Figure 5 and Figure 6, which depict the reduced viscosity vs. concentration graphs for two tacrolimus-polymer blends with different drug loadings (10–50%). In Figure 5, the tacrolimus-Eudragit^®^ S100 blends are shown, while in Figure 6, the blends of tacrolimus-Poloxamer-188 are given. Slope, intercept, and *R^2^* values for each tacrolimus-polymer combination are shown in Supplementary Materials (Table S1).

Miscibility parameters Δ*Β*, *μ*, and *α* were calculated for each tacrolimus-polymer combination, using the slope and intercept values derived from the reduced viscosity vs. concentration graphs. Parameters Δ*B* and *μ* are included in the Supplementary Materials (Table S2), while parameter α, as more discriminatory, is depicted in Table 5. Parameter *α* was found positive for four out of six tacrolimus-polymer combinations across the drug loadings tested, which suggests that these combinations are miscible. However, for the tacrolimus-PEG 6000 and the tacrolimus-Poloxamer-188 combinations, parameter α was found negative, suggesting immiscibility.

## 4. Discussion

The selection of miscible API-polymer combinations is of utmost importance for the preparation of successful ASDs. However, current screening approaches for selection of appropriate polymers are often tedious, cost intensive, and/or prone to theoretical limitations. In this work, we introduce an easy to apply, inexpensive method which is based on the dilute solution rheology theory for the investigation of miscibility between polymers and drugs, using tacrolimus as model API. The results of the novel approach are compared with in silico calculation of HSPs, as well as with monitoring of potential drug crystallization in the solid dispersions upon storage.

Typically, API-polymer miscibility is investigated by the preparation of solid dispersions in varying drug loading ratios. Such experiments can be run via a design of experiment (DoE) setup, where samples are tested over time regarding the lack of phase separation and/or API crystallinity [63]. Techniques such as DSC, XRPD, infrared and Raman spectroscopy, solid-state nuclear magnetic resonance, and scanning electron microscopy can be used to confirm and monitor the physical state of an API formulated as ASD [64,65,66,67]. Re-crystallization of amorphous API in candidate formulations is not uncommon and is a typical consequence of a phase separation [22]. Drug dispersion and solubility in a polymer matrix are linked properties, as both are driven by the molecular interaction forces. Once a phase separation occurs, there are regions formed of low and high drug concentrations relative to polymer. Especially the drug-rich domains in the matrix are likely to exhibit crystallization [68]. Consequently, the absence of detectable crystallization of the API in short-term stability testing may not guarantee an adequate shelf-life stability. Therefore, it is useful to identify sensitive drug-polymer mixtures regarding physical instability.

Despite the common use of XRPD and DSC to detect crystalline drug [69], one should keep in mind the analytical limitations. DSC temperature ramps may cause recrystallization of amorphous materials or, alternatively, increased temperature may lead to dissolution of crystalline drug in the polymer matrix, so the result may not necessarily reflect otherwise isothermal stability conditions over time [69,70]. In addition, the presence of excipients can introduce further limitations in the detection of the thermal behavior of the API due to overlapping signals [71,72]. On the other hand, XRPD has limitations concerning preferred crystal orientation due to morphology and/or processing parameters such as compression [69,73], and overall sensitivity of common laboratory equipment does not match the sensitivity in detecting crystalline material by using a synchrotron radiation. Despite these limitations, XRPD and DSC are still widely used to monitor amorphous formulations, as the analytical sensitivity is often good enough for most practical purposes and because of their simplicity in acquiring and interpreting data.

Our findings suggest an increased sensitivity in the detection of crystalline tacrolimus via XRPD compared to DSC, based on the identification of crystalline drug at time zero via XRPD but not DSC in the cases of tacrolimus mixtures with PEG 6000 and Poloxamer-188. This confirms earlier findings that demonstrated that the sensitivity of XRPD in detecting trace crystallinity in tacrolimus solid dispersions was greater than that of DSC [71].

The analytical limitations concerning the detection of crystalline tacrolimus were investigated also in a study by Purohit et al. [74], where it was noted that when tacrolimus capsules were left open in a high humidity environment (for tacrolimus to crystallize), XRPD showed a lag time in detecting crystalline tacrolimus. They attributed this either to the slow crystallization of tacrolimus or to the high amount of crystalline drug needed for XRPD to identify and quantify crystallinity traces [74]. Consequently, the presence of tacrolimus crystallinity in the 90%, but not the 10%, drug-loaded solid dispersions of drug with PEG 6000 and Poloxamer-188 at time zero could be attributed to low sensitivity of crystalline tacrolimus detection. This was further supported when considering that the aforementioned 10% drug-loaded combinations showed tacrolimus crystallinity later at 1 and 3 months. Therefore, such aspects of analytical sensitivity must be kept in mind when no crystalline drug is evident in drug-polymer mixtures. Such short-term monitoring of physical stability might be better used as complementary data on possible consequences of miscibility, or lack thereof. Especially for larger molecule combinations, demixing may be retarded, as it is a diffusion-mediated process [75]. This means that even though it is more difficult for larger molecules to mix on a molecular level, the increased viscosity due to large molecular size would likely slow down the demixing process.

Compared to any experimental monitoring over time, it is attractive to use in silico techniques for the assessment of miscible API-polymer combinations in ASDs, such as the calculation of solubility parameters. However, the strength, as well as the main weakness, of any solubility parameter approach is simplicity. For example, since the solubility parameter is a solution property, an application to solids comes naturally with assumptions. An application to high-energy solids like amorphous compounds [76] can be viewed similar to a supercooled liquid, but it is only an approximation [36]. In addition, it has been highlighted that the HSP calculations lack more detailed thermodynamic and, specifically, entropic considerations [36,37,38]. It is interesting to mention in the context of entropy, that the prediction of solubility in solvents via the original experimental Hansen method [58] seems to be more accurate for larger molecules, such as in paints and polymer mixtures [37] than using it for small-molecular APIs [77] in pharmaceutics and cosmetics [78]. This is due to smaller entropy gain from the dissolution of bigger molecules, so that a focus on enthalpic interactions by comparing HSPs becomes a more viable approach to miscibility or solubility. However, there have been proposals for thermodynamic improvements to the Hildebrand [79] and Hansen solubility parameter calculations [37].

The general difficulties in estimating drug-polymer miscibility in silico prompted the exploration of novel screening methods. In this work, the viscosity of tacrolimus-polymer blends was studied and modeled to acquire interaction parameters that can reveal miscibility trends for the API and the polymer. More specifically, the interaction parameters Δ*B*, *μ*, and *α* are indicators of miscibility when they take positive values, and indicators of immiscibility when they get negative, with the parameter *α* holding more predictive power [47,48,49]. From an experimental viewpoint, it makes sense to distinguish between mixtures in which electrostatic interactions occur as compared to other neutral systems. The presence of ionic interactions has been known to possibly lead to complex behavior, where reduced viscosity can even increase with decreasing concentrations [80]. Already, Chee mentioned that the rheology-based method for the assessment of miscibility works better for non-electrolyte solutions [47]. This means that the rheology-based results should be interpreted with care in electrolyte solutions. In our study, Eudragit^®^ S100 was the only excipient with ionizable groups, and results from dilute solution viscosimetry were in line with calculated HSP, as well as the physical characterization of the tested solid dispersions. Since ethanol has a considerably lower dielectric constant compared to that of water (i.e., about 20 vs. 80 at 20 °C) [81], electrostatic interactions in the ethanol tacrolimus-Eudragit^®^ S100 solutions should be minimal. Therefore, it is advantageous to select less polar solvents for the rheological method, not only to assure sufficient solubility of the compound, but also to avoid artefacts from pronounced ionization.

Overall, our findings suggest that there is a fair consensus regarding the miscible tacrolimus-polymer combinations. Four out of the six combinations tested were found to be miscible from rheology, which was in good agreement with calculated HSPs and stability experiments that did not reveal drug re-crystallization. The data of the different methods also agreed that the tacrolimus-PEG 6000 combinations were immiscible. Different was the case of tacrolimus-Poloxamer-188 combinations, which were deemed immiscible based on the rheological approach and with the physical monitoring of the formulations, but this was not predicted by the in silico calculation of the HSPs (Table 6).

**Figure 7:**
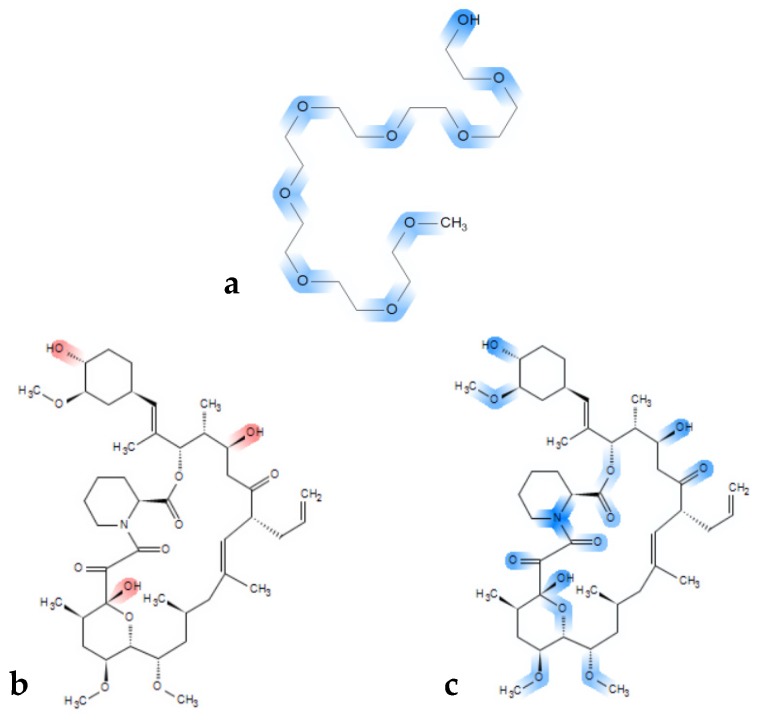
Chemical structure of (**a**) a PEG model chain with marked blue hydrogen bond acceptor sites and (**b**) chemical structure of tacrolimus with marked red hydrogen donor moieties and (**c**) with acceptor sites marked in blue.

The rather clear immiscibility outcome for the tacrolimus-PEG 6000 combinations is of importance, as PEGs are widely used polymers in ASD formulations, because of their solubility in water and their low cost [82,83]. It has also been proposed that the success of PEG as a carrier in ASDs depends on the physicochemical characteristics of the API used, the interactions between the polymer and the API [84,85], as well as the molecular weight of PEG [86]. PEG has two hydrogen donor sites on both ends and multiple acceptor sites depending on the molecular weight of the polymer (ether groups) (Figure 7a). Consequently, in higher molecular weight PEG polymers, more hydrogen bond acceptor sites exist, and the effect of the hydroxyl groups that act as hydrogen donor sites can be considered as minimal. Thus, despite tacrolimus exhibiting eleven hydrogen acceptor and three hydrogen donor sites (Figure 7b red markings, and Figure 7c blue markings), it might be not ideal for hydrogen bond formation with PEG. Moreover, high molecular weight PEG generally entails higher crystallinity, given that it is a semi-crystalline polymer, which is unfavorable for miscibility in general [87,88,89].

Poloxamer-188 is also a semi-crystalline polymer that was used in this study. Tacrolimus-Poloxamer-188 combinations was deemed immiscible based on physical state monitoring and the rheology-based technique, but not the HSP calculations. Immiscibility could be expected for these combinations due to the semi-crystalline nature of the polymer, as well as due to the structural similarity of Poloxamer-188 and PEG, which was deemed generally immiscible with tacrolimus. Given that it is highly unlikely that Poloxamer-188 and tacrolimus are miscible, the inability of the HSPs to reveal the immiscibility between Poloxamer-188 and tacrolimus could be attributed to different reasons, but most relevant were limitations considering partially and entirely crystalline materials [39] and the amphiphilic character of Poloxamer-188.

**Figure 8:**
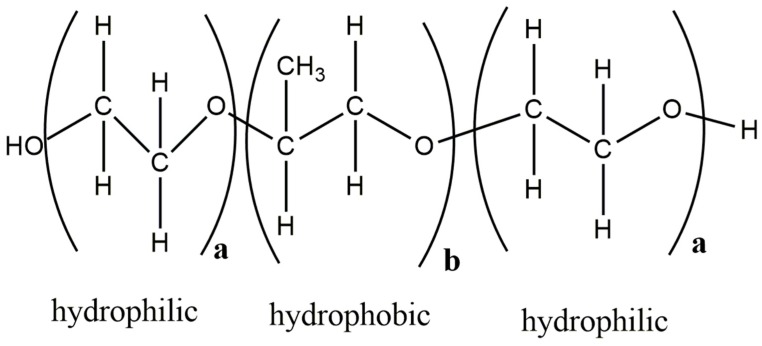
Structure of Poloxamer-188 with hydrophobic and hydrophilic units, (adapted from [90]). The figure was prepared with ChemDraw Version 19.0 software (PerkinElmer Informatics, Inc., Walthman, MA, USA).

Poloxamer-188 is a triblock copolymer and is made of units with different characteristics, i.e., having two hydrophilic and one hydrophobic domain (Figure 8). It can be well imagined that for such amphiphilic cases, the conventional HSP calculation is problematic as it can only account for a homogenous systems and not for different molecular environments in the same polymer matrix. There has been recent progress in capturing the HSP of block copolymers. An approach where experimental solubility data need to be combined with a double Hansen solubility sphere is proposed in such cases [59].

In the present study, the miscibility of tacrolimus was investigated with another copolymer, Soluplus^®^. This copolymer was found to be miscible with the drug in accordance with the results of the different approaches, including the HSP calculations. Contrary to the semi-crystalline Poloxamer-188, Soluplus^®^ is an amorphous copolymer. Amorphous carriers have also been shown to improve crystallization inhibition and amorphization capacity compared to crystalline and semi-crystalline polymers [91]. This is due to the large amorphous domains within the polymer and the increased viscosity of these polymers at room temperature that restricts the drug motion and diffusion, and thus its crystallization [92]. Moreover, as mentioned previously, the HSP calculations approximate an amorphous solid state as a supercooled liquid, which would make the predictions for Soluplus^®^ more accurate than that of the partially crystalline Poloxamer-188.

It seems that currently, all approaches to screen drug-polymer miscibility have their advantages and limitations. In the case of the novel rheological method, a main advantage is that it offers a theory-based approach on screening for miscible API-polymer combinations, when dealing with APIs that exhibit higher molecular weight than the most currently druggable APIs. This is becoming a pressing need, as there is a well-documented increase in the molecular weight of commercially available formulated APIs, with their molecular weight increasing on average about 1 g/mol per year [1]. In addition, this method exhibits a much faster and less tedious experimental determination compared to HSP or the χ parameter. The experimental part of the rheological method makes it, further, more reliable as compared to any in silico estimation of an HSP or a χ parameter. However, drawbacks of the rheological method include the need of a common solvent for the components and, in the case of pronounced ionization in the given solvent, the method can become less reliable. In general though, despite its limitations, the rheology-based method implemented here offers an attractive alternative to the already existing techniques to investigate API-polymer miscibility.

## 5. Concluding Remarks

Adequate miscibility between API and stabilizing polymer is a prerequisite for successful formulation of ASDs. However, currently employed methods for the screening of miscible combinations show limitations. There is, presently, research invested to use modern thermodynamic approaches beyond the HSP and Flory Huggins χ parameter, but there is still much parameterization work needed for any practical implementation. As an in silico method, calculation of HSPs can still be viewed as a simple and useful method. Results of this study show that HSP calculations were useful in many cases, but there was also overestimation for some polymers to be miscible with the model drug tacrolimus. The novel rheology-based method provided here a valuable complementation of the data as it was fast, inexpensive, and analytically robust. Based on the underlying theory and the results, this method is especially suitable for APIs exhibiting bRo5 characteristics, such as for tacrolimus. Future research will show how broadly the novel approach can be implemented in both academia, as well as the pharmaceutical industry.

## Figures and Tables

**Table 1:** Physicochemical characteristics of the compounds used in this study.

Compound	Molecular Weight (Da)	Melting Temperature (°C)	Glass Transition Temperature (°C)
Tacrolimus	804.018	126	78.8
HPC-L	95,000	-	105
EC	-	240–255	140
Soluplus^®^	118,000	-	70
PEG 6000	6000	58–63	-
Poloxamer-188	8500	52	−22
Eudragit^®^ S100	12,500	188	125–135

**Table 2:** Stability of amorphous tacrolimus in solid dispersions with the polymers used in this study. Solid dispersions were prepared with the rotary evaporator and by film casting. The amorphous state of tacrolimus was investigated with DSC and XRPD at time zero and with DSC at the time points of 1 and 3 months. A indicates that tacrolimus is deemed amorphous and ***C*** indicates that tacrolimus is deemed crystalline.

Polymer	Drug Loading	Rotary Evaporator				Film Casting			
		Time Zero		1 Month	3 Months	Time Zero		1 Month	3 Months
		XRPD	DSC	DSC	DSC	XRPD	DSC	DSC	DSC
HPC-L	10%	A	A	A	A	A	A	A	A
	90%	A	A	A	A	A	A	A	A
EC	10%	A	A	A	A	A	A	A	A
	90%	A	A	A	A	A	A	A	A
Soluplus^®^	10%	A	A	A	A	A	A	A	A
	90%	A	A	A	A	A	A	A	A
PEG	10%	A	A	***C***	***C***	A	A	***C***	***C***
	90%	***C***	A	***C***	***C***	***C***	A	***C***	***C***
Poloxamer	10%	A	A	***C***	***C***	A	A	***C***	***C***
	90%	***C***	A	***C***	***C***	***C***	A	***C***	***C***
Eudragit^®^ S100	10%	A	A	A	A	A	A	A	A
	90%	A	A	A	A	A	A	A	A

**Table 3:** Hansen solubility parameter (HSP) values for the compounds used in this study, as calculated by the HSPiP software. In the final column, there is the outcome of miscibility prediction between each polymer and tacrolimus.

Compound	*δ_d_* (MPa^0.5^)	*δ_p_* (MPa^0.5^)	*δ_h_*(MPa^0.5^)	*δ_t_* (MPa^0.5^)	|Δ*δ_t_* = *δ_tac_ − δ_pol_*| (MPa^0.5^)	Outcome
Tacrolimus	18.8	3.1	5	19.7	N/A	N/A
HPC-L	16.8	8.5	1.9	19	0.7	Miscible
EC	16	7.8	6.8	19	0.7	Miscible
Soluplus^®^	17.4	6.2	8.7	20.4	0.7	Miscible
PEG 6000	17.8	13.5	27.4	35.3	15.6	Immiscible
Poloxamer-188	16.4	6.9	5.8	18.7	1	Miscible
Eudragit^®^ S100	16	4.4	8.7	18.7	1	Miscible

**Table 4:** *R_a_* and relative energy difference (RED) values for the compounds used in this study, as calculated by the HSPiP software. In the final column, there is the outcome of miscibility prediction between each polymer and tacrolimus.

Compound	*R_a_*	RED = *R_a_/R*_0_ *R*_0_ = 22 MPa^0.5^	Outcome
Tacrolimus	N/A	N/A	N/A
HPC-L	7.4	0.33	Miscible
EC	7.5	0.34	Miscible
Soluplus^®^	5.5	0.25	Miscible
PEG 6000	24.8	1.12	Immiscible
Poloxamer-188	6.2	0.28	Miscible
Eudragit^®^ S100	6.8	0.31	Miscible

**Table 5:** Miscibility parameter α of tacrolimus and polymer blends in different drug loadings. Positive values with the (+) sign indicate miscibility while negative values with the (−) sign indicate immiscibility.

Polymer	Drug Loading	*α*
HPC-L	10%	2.67 (+)
	25%	1.37 (+)
	50%	0.71 (+)
EC	10%	6.27 (+)
	25%	3.20 (+)
	50%	0.40 (+)
Soluplus^®^	10%	0.08 (+)
	25%	0.14 (+)
	50%	0.47 (+)
PEG 6000	10%	−0.65 (−)
	25%	−0.45 (−)
	50%	−0.77 (−)
Poloxamer-188	10%	−2.38 (−)
	25%	−3.38 (−)
	50%	−5.32 (−)
Eudragit^®^ S100	10%	2.40 (+)
	25%	3.65 (+)
	50%	1.71 (+)

**Table 6:** Comparison of miscibility data from the two methods investigated in this study, accompanied by the complimentary data of the physical state monitoring technique.

Polymer	Physical State after 3 Months		In Silico Method	Rheology Method	
	Drug Loading	Outcome		Drug Loading	Outcome
HPC-L	10%	Amorphous	Miscible	10%	Miscible
				25%	
	90%			50%	
EC	10%	Amorphous	Miscible	10%	Miscible
				25%	
	90%			50%	
Soluplus^®^	10%	Amorphous	Miscible	10%	Miscible
				25%	
	90%			50%	
PEG 6000	10%	Crystalline	Immiscible	10%	Immiscible
				25%	
	90%			50%	
Poloxamer-188	10%	Crystalline	Miscible	10%	Immiscible
				25%	
	90%			50%	
Eudragit^®^ S100	10%	Amorphous	Miscible	10%	Miscible
				25%	
	90%			50%	

## Supplementary Materials

The following are available online at https://www.mdpi.com/1999-4923/11/12/625/s1, Figure S1: DSC thermographs for Ethylcellulose (EC) (a), 10% tacrolimus:EC (b), and 90% tacrolimus:EC (c), Figure S2: XRPD spectra of crystalline tacrolimus (a), Ethylcellulose (EC) (b) and of fresh tacrolimus formulations prepared via the film casting method of 90% tacrolimus:EC (c) and 90% tacrolimus:EC (d). Figure S3: XRPD spectra of crystalline tacrolimus (a), Poloxamer-188 (b) and PEG 6000 (c) and of fresh tacrolimus formulations prepared via the film casting method of 90% tacrolimus:Poloxamer-188 (d) and 90% tacrolimus:PEG 6000 (e), Table S1: Slope, intercept and R^2^ values derived from the reduced concentration vs. concentration graphs for all tacrolimus:polymer combinations, Table S2: Slope, intercept and R^2^ values derived from the reduced concentration vs. concentration graphs for all tacrolimus:polymer combinations.
