# Supplementary material for: A Novel Rheological Method to Assess Drug-Polymer Interactions Regarding Miscibility and Crystallization of Drug in Amorphous Solid Dispersions for Oral Drug Delivery
Source: Pharmaceutics. 2019 Nov 22;11(12):625. doi: 10.3390/pharmaceutics11120625 (PMC6955678; doi:10.3390/pharmaceutics11120625)
Supplement: Supplementary file 1 [file pharmaceutics-11-00625-s001.pdf]

pharmaceutics

Supplementary Materials: A Novel Rheological Method to Assess Drug-Polymer Interactions Regarding Miscibility and Crystallization of Drug in Amorphous Solid Dispersions for Oral Drug Delivery

Georgia Tsakiridou, Christos Reppas, Martin Kuentz and Lida Kalantzi

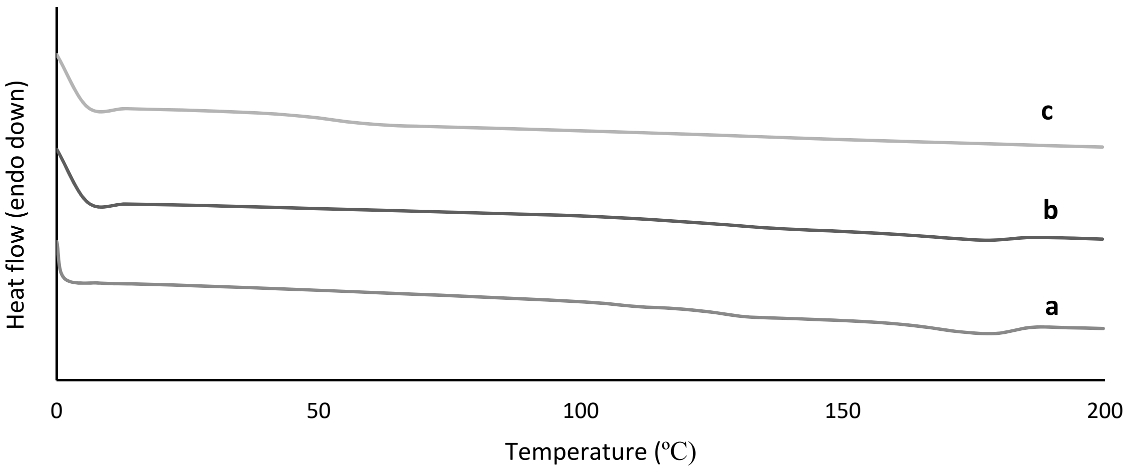

Figure S1. DSC thermographs for Ethylcellulose (EC) (a) 10% tacrolimus:EC, (b) and 90% tacrolimus:EC (c).

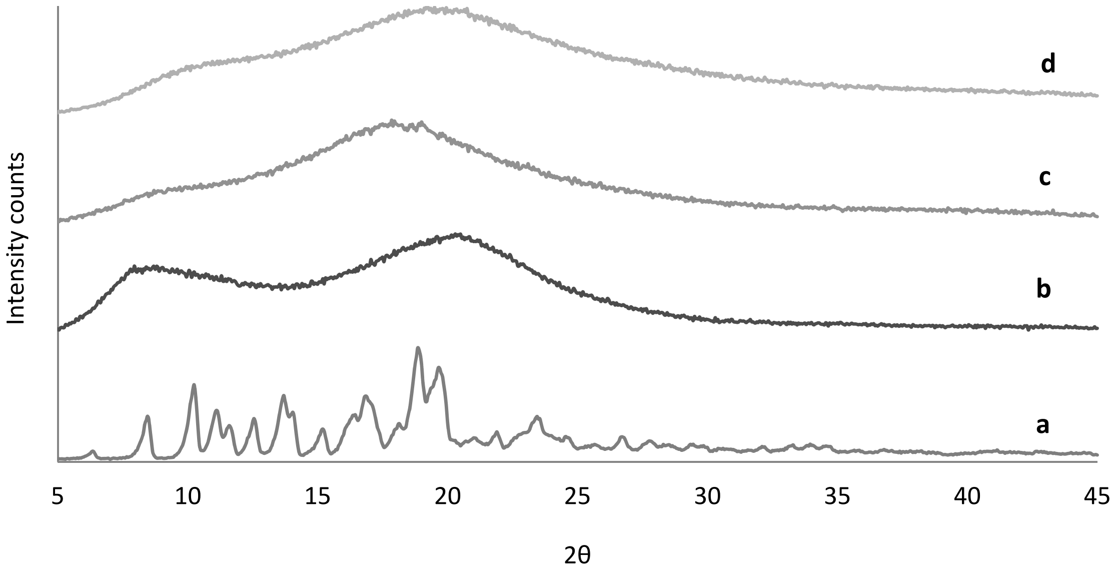

Figure S2. XRPD spectra of crystalline tacrolimus (a), Ethylcellulose (EC) (b) and of fresh tacrolimus formulations prepared via the film casting method of 90% tacrolimus:EC (c) and 90% tacrolimus:EC (d).

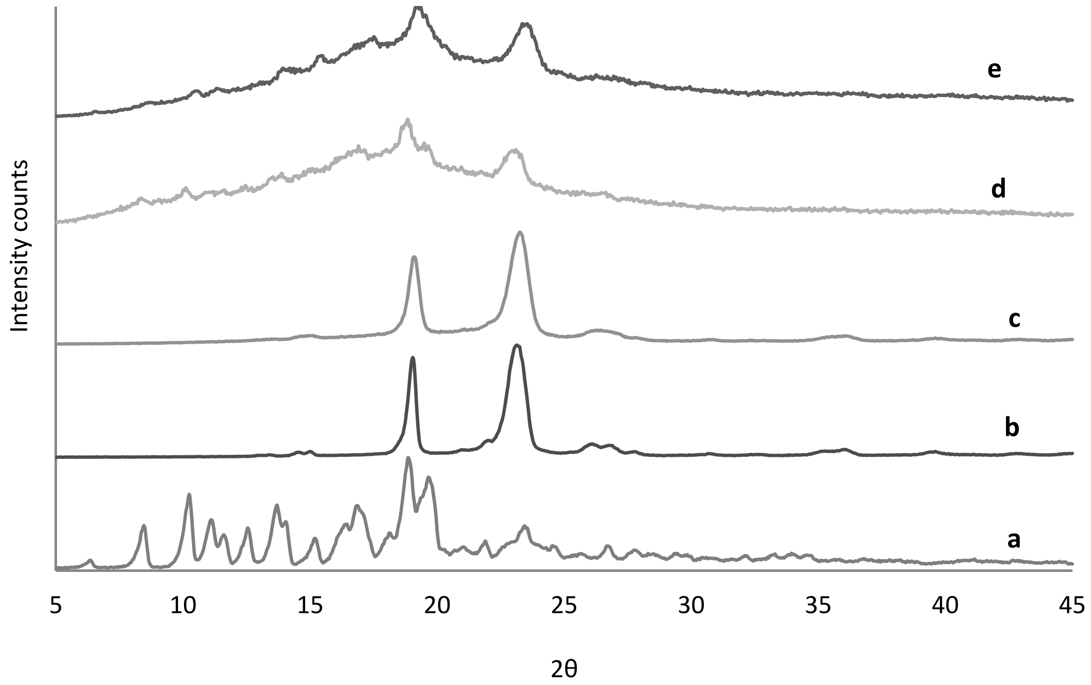

Figure S3. XRPD spectra of crystalline tacrolimus (a), Poloxamer-188 (b) and PEG 6000 (c) and of fresh tacrolimus formulations prepared via the film casting method of 90% tacrolimus:Poloxamer-188 (d) and 90% tacrolimus:PEG 6000 (e).

Table S1. Slope, intercept and R^2 values derived from the reduced concentration vs. concentration graphs for all tacrolimus:polymer combinations.

Polymer	Drug Loading	Slope	Intercept	R^2 value
Tacrolimus	N/A	0.22	0.12	0.93
HPC-L	10%	1.75	0.46	0.98
	25%	1.95	0.47	0.93
	50%	2.98	0.49	0.97
EC	10%	0.42	0.21	0.97
	25%	0.66	0.30	0.95
	50%	0.64	0.30	0.97
Soluplus®	10%	1.07	1.25	0.92
	25%	1.01	1.01	0.84
	50%	1.02	0.72	0.97
PEG 6000	10%	1.73	1.87	0.73
	25%	3.57	2.08	0.70
	50%	5.10	2.38	0.69
Poloxamer-188	10%	1.06	1.37	0.65
	25%	1.88	1.57	0.56
	50%	4.78	1.76	0.65
Eudragit® S100	10%	0.76	0.45	0.95
	25%	0.61	0.33	0.98
	50%	0.64	0.35	0.92

Table S2. Calculated ΔB and μ miscibility parameters for all tacrolimus:polymer combinations used in this study.

Polymer	Drug Loading	ΔB	μ
HPC-L	10%	0.06 (+)	0.22(+)
	25%	0.06 (+)	0.23 (+)
	50%	−0.22 (−)	−0.77 (−)
EC	10%	0.03 (+)	0.08 (+)
	25%	0.02 (+)	0.08 (+)
	50%	−0.05 (−)	−0.16 (−)
Soluplus®	10%	0.03 (+)	0.02 (+)
	25%	0.06 (+)	0.04 (+)
	50%	0.07 (+)	0.05 (+)
PEG 6000	10%	0.27 (+)	0.04 (+)
	25%	−0.23 (−)	−0.03 (−)
	50%	−0.50 (−)	−0.07 (−)
Poloxamer-188	10%	0.03 (+)	0.10 (+)
	25%	−0.05 (−)	−0.14 (−)
	50%	−0.31 (−)	−0.92 (−)
Eudragit® S100	10%	0.02 (+)	0.04 (+)
	25%	0.04 (+)	0.07 (+)
	50%	0.05 (+)	0.08 (+)
